# Effects of recombinant human soluble thrombomodulin on neutrophil extracellular traps in the kidney of a mouse model of endotoxin shock

**DOI:** 10.20407/fmj.2022-026

**Published:** 2022-12-27

**Authors:** Tatsuhiko Harada, Yasuyo Shimomura, Osamu Nishida, Munenori Maeda, Yu Kato, Tomoyuki Nakamura, Naohide Kuriyama, Hidefumi Komura

**Affiliations:** Department of Anesthesiology and Critical Care Medicine, Fujita Health University, School of Medicine, Toyoake, Aichi, Japan

**Keywords:** Neutrophil extracellular trap, Sepsis, Thrombomodulin, Lipopolysaccharide, Kidney

## Abstract

**Objectives:**

Sepsis is a life-threatening condition characterized by multi-organ dysfunction due to host immune system dysregulation in response to an infection. During sepsis, neutrophils release neutrophil extracellular traps (NETs) as part of the innate immune response. However, excessive NETs play a critical role in the development of organ failure during sepsis. Although recombinant human soluble thrombomodulin (rTM) can inhibit NET formation in the lungs and liver of a mouse model of endotoxin shock, its effects on the kidneys are unclear.

**Methods:**

The specific effects of NETs and rTM on the renal cortex and renal medulla were examined in a mouse model of endotoxin shock generated by intraperitoneal (i.p.) injection of lipopolysaccharide (LPS), followed by i.p. injection of rTM or an identical volume of saline 1 h later.

**Results:**

LPS injection increased serum creatinine, blood urea nitrogen, and histone H3 levels. However, rTM administration significantly decreased histone H3 and citrullinated histone H3 (citH3) levels. Immunohistochemical analysis revealed no significant changes in citH3 quantity in the renal cortex of any group. However, in the renal medulla, the increase in citH3 induced by LPS was abolished in the LPS+rTM group.

**Conclusions:**

Our findings demonstrate that rTM can suppress NETs in the renal medulla of mice with endotoxin-induced acute kidney injury.

## Introduction

Sepsis, a type of multi-organ dysfunction, results from infection-induced dysregulation of immune responses.^1^ A recent study revealed that hospital mortality rates associated with sepsis and severe sepsis in the United States are 17% and 26%, respectively.^2^ In addition, sepsis can cause acute kidney injury (AKI),^3,4^ and considerably increases the duration of hospital stays and in-hospital mortality rates compared with non-septic AKI.^5^ However, the pathophysiology of AKI in sepsis remains unknown, and better understanding could improve treatment strategies for sepsis.

As a part of the innate immune response, neutrophils release substances such as histones and nuclear DNA, which form reticular structures called neutrophil extracellular traps (NETs).^6^ As these reticular structures trap and kill bacteria,^7^ they affect coagulation and thrombogenesis.^8,9^ NET formation contributes to immune thrombosis during the pathogenesis of sepsis.^9^ However, excessive NET formation can lead to capillary circulation disorders.^10,11^ During sepsis, rapid systemic inflammation causes these immune responses to affect both infected and non-infected regions of the body, leading to multiple-organ dysfunction.^12^ NETs release citrullinated histones, which induce platelet aggregation^13,14^ and inflammation.^15^ Direct histone injection into the kidney has been shown to cause neutrophil migration, microvascular leakage, and renal inflammation.^16^

Thrombomodulin (TM) is an important cofactor in the anticoagulant pathway that exerts anticoagulation effects *in vivo*, however, its expression is decreased during the development of septic-disseminated intravascular coagulation.^17^ Recombinant human soluble TM (rTM) can inhibit NET formation by binding to histones.^18^ Previously, we reported that injecting rTM into a mouse model of endotoxin shock improved the survival rate and inhibited NET formation in both lung and liver tissues.^19^ However, the effects of rTM on the kidney remain controversial. Hayase et al. found that rTM administration inhibited histone accumulation and NET formation in the lungs, but not kidneys, of a mouse model of renal ischemia-reperfusion injury.^20^ In contrast, Nozaki et al. and Akatsuka et al. reported that rTM reduces renal damage.^21,22^ The kidney has multiple physiological roles: the glomerulus and glomerular capillary produce primary urine in the renal cortex, whereas the nephron and peritubular capillary reabsorb nutrients from primary urine in the renal medulla. Notably, previous studies examined the kidney as a single organ, with no distinction made between the renal cortex and renal medulla. The immune function of healthy renal medulla is more developed than that of the renal cortex, as antigen-presenting dendritic cells are more prevalent in the peritubular interstitium.^23^ Moreover, the high-sodium environment of the renal medulla induces chemokines that promote immune function.^24^ We suspected that the renal medulla plays an important role in the immune response during sepsis as a result of its well-developed immune function. Therefore, we hypothesized that NETs are abundant in the renal medulla of endotoxin-shocked mice, and rTM can effectively reduce NETs. In this study, we aimed to evaluate the specific effects of rTM on the renal cortex and renal medulla in an endotoxin shock mouse model.

## Methods

### Animals and study design

Female C57BL/6J Jms mice were obtained from SLC (Hamamatsu, Japan) and maintained in our conventional mouse and rat facility with free access to food and water. Mice aged 6–8 weeks were used after at least 6 days of acclimation. Lipopolysaccharide (LPS; 125-05201, Wako Pure Chemical Industries, Osaka, Japan) and rTM (ART-123; Asahi Kasei Pharma, Tokyo, Japan) were used in the present study. To create the endotoxin shock model, 10 mg/kg LPS was intraperitoneally (i.p.) injected into mice. One hour after LPS injection, 6 mg/kg rTM (rTM group) or an equal volume of saline (non-rTM group) was administered (i.p.). Control mice were injected with an identical volume of saline instead of LPS or rTM. Eight hours after LPS/saline injection, mice were anesthetized with isoflurane and euthanized by cardiac puncture. Blood and kidney samples were collected for analysis. Symptoms of sepsis, such as lethargy, piloerection, decreased appetite, and a hunched position, were also observed.

### Ethics statement

All mice were handled according to Regulations for the Management of Laboratory Animals at Fujita Health University (Toyoake, Japan). All animal protocols were approved by the Animal Care and Use Committee of Fujita Health University (Approval No. APU19079-MD1, 5 January 2022). The point when mice displayed loss of the righting reflex while in a dorsal position was classified as the humane endpoint, at which point mice were culled.

### Measurement of histone H3 (H3) and citrullinated H3 (citH3) levels

Serum was separated from collected blood samples by centrifugation at 1470 × g for 10 min at 4°C. Serum H3 levels were measured using enzyme-linked immunosorbent assay (ELISA) with human anti-H3 antibodies (Shino-Test Corporation, Sagamihara, Japan) as previously described.^25^ citH3 was detected using an ELISA kit (501620; Cayman Chemical, Ann Arbor, MI, USA).

### Measurement of blood creatinine (CRE) and blood urea nitrogen (BUN) levels

Dri-CHEM (NX600; Fujifilm, Tokyo, Japan) was used to measure CRE (v-CRE-P, Fujifilm) and BUN (v-BUN-P, Fujifilm) levels. Slides were placed in the machine and 10 μL of serum was dropped onto the slides. Spotting was carried out manually using a pipette because the sample volume was small.

### Immunofluorescence staining

Excised kidneys were immediately fixed in 4% paraformaldehyde phosphate buffer solution (Fujifilm), and 3-μm-thick sections were prepared after paraffin embedding. Sections were incubated with primary antibodies against lymphocyte antigen 6 complex locus G6D (Ly-6G; 551459; BD Bioscience, Franklin Lakes, NJ, USA) and histone H3 (citrulline R2+R8+R17, citH3; ab5103; Abcam, Cambridge, UK), followed by Alexa Fluor 488- (ab172332, Abcam) and Alexa Fluor 594-conjugated (A21207; Thermo Fisher Scientific, Waltham, MA, USA) secondary antibodies. Nuclei were stained using 4',6-diamidino-2-phenylindole dihydrochloride solution (DAPI; Thermo Fisher Scientific). Tissue sections were observed and imaged using a confocal laser-scanning microscope (LSM 980; Zeiss, Oberkochen, Germany).

### Quantification of citH3 under immunohistochemical staining

Stained kidneys were imaged at 400× magnification with an LSM 980 (Zeiss). Numbers of citH3-positive cells were quantified in the renal cortex and renal medulla. In the renal cortex, three images were taken per sample, citH3-positive cells in the glomerulus were counted, and the mean value was calculated. In the renal medulla, a single image was taken per sample and citH3-positive cells in the field of view were counted. Fiji (ImageJ, version 1.53q; http://imagej.nih.gov)^26^ was used for manual quantification.

### Statistical analysis

Serum CRE, BUN, H3, and citH3 levels were analyzed using the Mann–Whitney U test. Numbers of citH3-positive cells evaluated by immunohistochemical staining were analyzed using an unpaired t-test. Results with *P*<0.05 were considered statistically significant. All statistical analyses were conducted using GraphPad Prism version 9.3.1 (GraphPad Software, San Diego, CA, USA).

## Results

### Effects of rTM on Serum H3 and citH3 Levels during Endotoxin Shock

**Figure 1:**
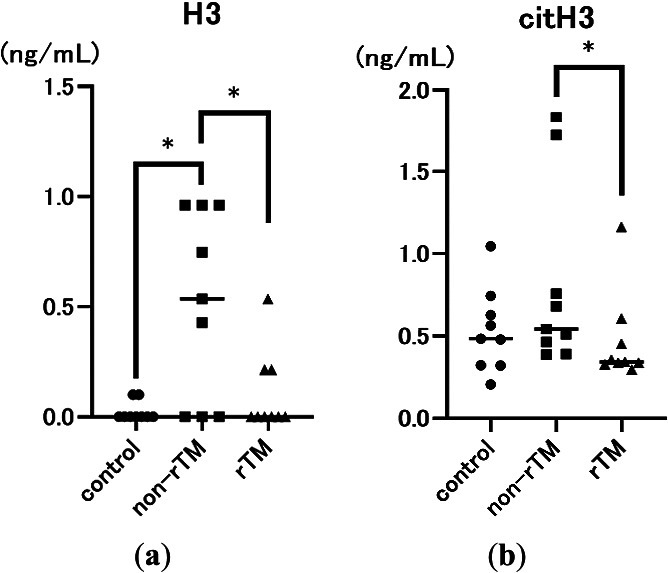
Effects of lipopolysaccharide (LPS) and recombinant human soluble thrombomodulin (rTM) on serum H3 (a) and citrullinated H3 (citH3) levels (b). Mice were intraperitoneally (i.p.) injected with 10 mg/kg LPS (non-rTM group; ■, *n*=9), followed by 6 mg/kg rTM after 1 h (rTM group; ▲, *n*=9). Control group mice (●, *n*=9) were i.p. injected with saline instead of LPS or rTM. Blood was collected 8 h after LPS injection. Data are expressed as the median. * *p*<0.05.

First, we examined the effects of rTM on serum H3 and citH3 levels in the mouse model 8 h after LPS injection. LPS significantly increased serum H3 levels (H3: 0.535 mg/dL, *p*=0.0202; citH3: 0.542 mg/dL) compared with the control (H3: 0.000 mg/dL; citH3: 0.484 mg/dL). However, rTM administration significantly decreased serum H3 and citH3 levels (H3: 0.000 mg/dL, *p*=0.0493; citH3: 0.342 mg/dL, *p*=0.0179; Figure 1) following LPS induction.

### Effects of rTM on blood CRE and BUN levels during endotoxin shock

**Figure 2:**
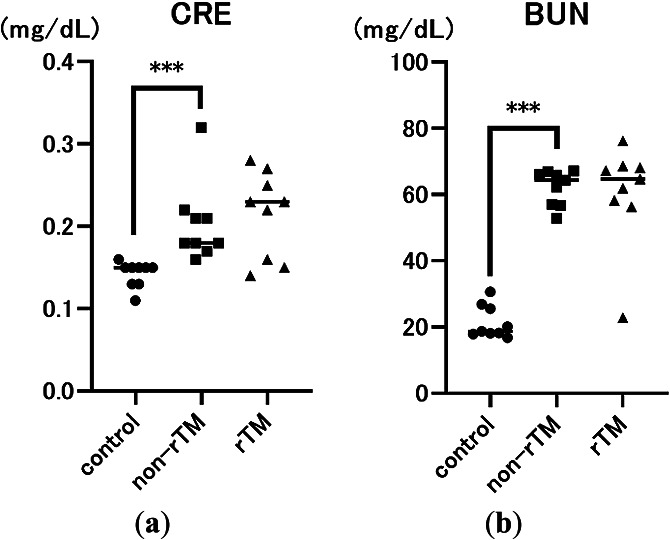
Effects of LPS and rTM on serum creatinine (CRE) (a) and blood urea nitrogen (BUN) levels (b). Mice were i.p. injected with 10 mg/kg LPS (non-rTM group; ■, *n*=9), followed by 6 mg/kg rTM after 1 h (rTM group; ▲, *n*=9). Control group mice (●, *n*=9) were i.p. injected with saline instead of LPS or rTM. Blood was collected 8 h after LPS injection. Data are expressed as the median. *** *p*<0.001.

To determine the effects of LPS and rTM, we also measured two indicators of endotoxin-induced renal injury, serum CRE and BUN levels, 8 h after LPS administration. As expected, CRE and BUN levels were significantly higher in the non-rTM group (CRE: 0.18 mg/dL, *p*<0.0001; BUN: 64.4 mg/dL, *p*<0.0001) compared with the control group (CRE: 0.15 mg/dL; BUN: 18.7 mg/dL). However, there was no significant difference in CRE or BUN levels between non-rTM and rTM groups (CRE: 0.23 mg/dL; BUN: 64.7 mg/dL, Figure 2).

### Effects of rTM on NETs in kidneys during endotoxin shock

**Figure 3:**
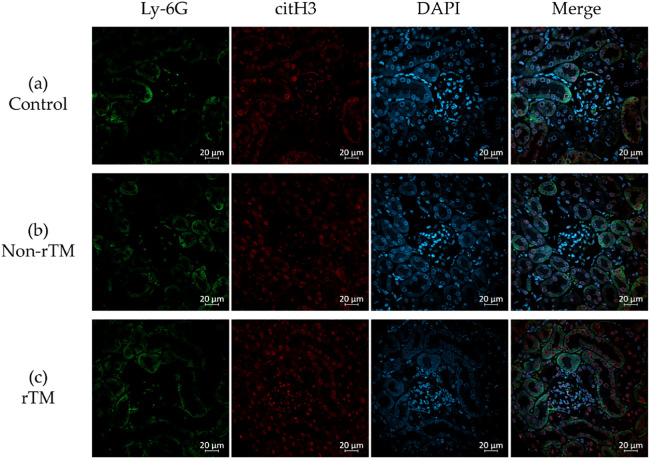
Inhibitory effects of rTM on neutrophil extracellular trap (NET) formation in the renal cortex. Mice were i.p. injected with 10 mg/kg LPS (non-rTM group), followed by 6 mg/kg rTM after 1 h (rTM group). Control group mice were i.p. injected with saline instead of LPS or rTM. The kidney was removed 8 h after LPS injection and subjected to immunofluorescence staining for Ly-6G, citH3, and DAPI. (a) Control, (b) LPS-injected, and (c) LPS- and rTM-injected renal cortex. Green, Ly-6G; red, citH3; blue, DAPI. Magnification: 400×, scale bar=20 μm.

**Figure 4:**
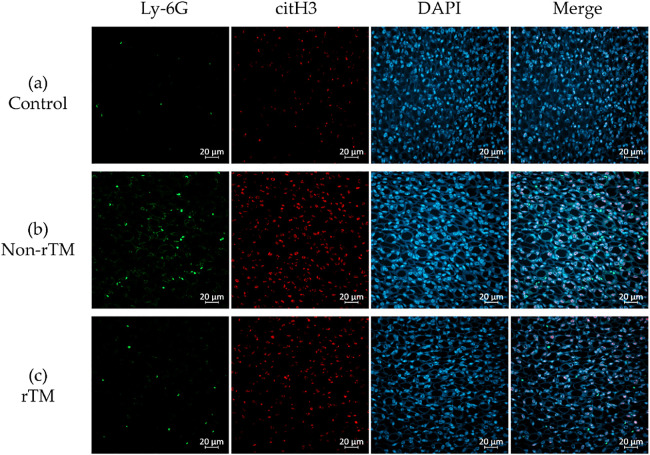
Inhibitory effects of rTM on NET formation in the renal medulla. Mice were i.p. injected with 10 mg/kg LPS (non-rTM group), followed by 6 mg/kg rTM after 1 h (rTM group). Control group mice were i.p. injected with saline instead of LPS or rTM. The kidney was removed 8 h after LPS injection and subjected to immunofluorescence staining for Ly-6G, citH3, and DAPI. (a) Control renal medulla; (b) LPS-injected renal medulla; (c) LPS- and rTM-injected renal medulla. Green, Ly-6G; red, citH3; blue, DAPI. Magnification: ×400, scale bar=20 μm.

**Figure 5:**
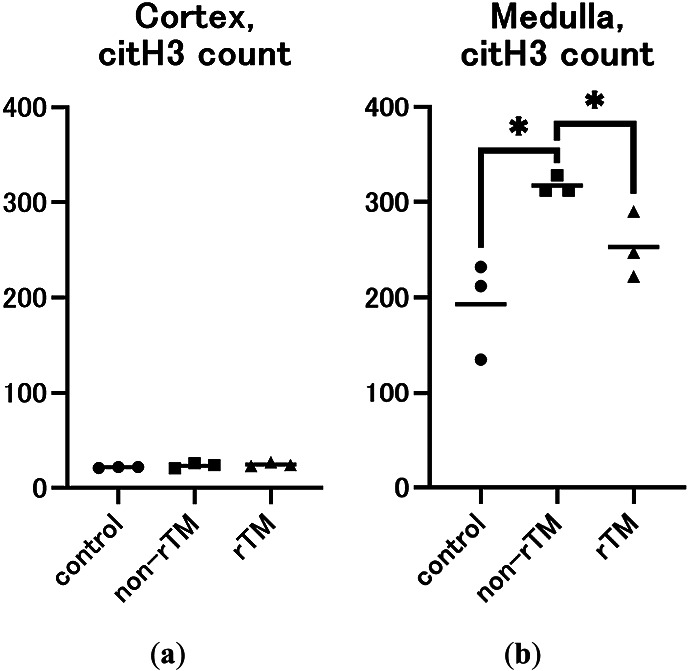
Numbers of citH3-positive cells in the renal cortex (a) and renal medulla (b). Mice were intraperitoneally (i.p.) injected with 10 mg/kg LPS (non-rTM group; ■, *n*=3), followed by 6 mg/kg rTM after 1 h (rTM group; ▲, *n*=3). Control group mice (●, *n*=3) were i.p. injected with saline instead of LPS or rTM. The kidney was removed 8 h after LPS injection and subjected to immunofluorescence staining for citH3. citH3-positive cells were counted manually using Fiji (ImageJ 1.53q)^26^ on a 400× magnified image. (a) In the renal cortex, three images were taken per sample, citH3-positive cells in the glomerulus were counted, and the mean was calculated. (b) In the renal medulla, a single image was taken per sample and citH3-positive cells in the field of view were counted. Data are expressed as the mean. * *p*<0.05.

To confirm the effects of rTM on NETs in the kidney, the left kidney was removed 8 h after LPS administration and subjected to immunofluorescence staining to identify neutrophils (Ly-6G, green) and NETs (citH3, red). Immunohistochemical analysis revealed no significant changes in citH3 in the renal cortex between control (21.87 count/glomerulus) and non-rTM groups (23.57 count/glomerulus), or between non-rTM and rTM groups (24.77 count/glomerulus, Figures 3 and 5). In the renal medulla, citH3 was increased in the non-rTM group (317.3 count/HPF, *p*=0.0144) compared with the control group (193.0 count/HPF) and decreased in the rTM group (253.0 count/HPF, *p*=0.0352, Figures 4 and 5). Notably, citH3 luminescence was observed in both the renal cortex and renal medulla of the control group, suggesting the possibility of autoluminescence.

## Discussion

Sepsis is a life-threatening condition characterized by multi-organ dysfunction due to dysregulation of the host immune system in response to an infection.^1^ During sepsis, neutrophils release NETs as part of the innate immune response, which can trap pathogens and prevent them from spreading. However, an excessive NET response can induce microvascular occlusion and tissue damage.^8,27^ Previously, we reported that rTM inhibits LPS-induced NETs *in vitro* in human neutrophils and platelets,^28^ and rTM injection can decrease lethality in mice with endotoxin shock to inhibit NET formation in the lungs and liver.^19^ However, the effects of rTM on the kidneys remain unclear. Because the kidney has various physiological roles, we evaluated the specific effects of rTM on the renal cortex and renal medulla of mice with endotoxin shock. First, we confirmed that LPS significantly increased serum CRE, BUN, and H3 levels compared with the control, suggesting that LPS successfully induced AKI in mice (Figures 1a and 2). In addition, we found that rTM significantly decreased serum H3 and citH3 levels in LPS-treated mice, suggesting that it effectively alleviated endotoxin shock (Figure 1).

**Figure 6:**
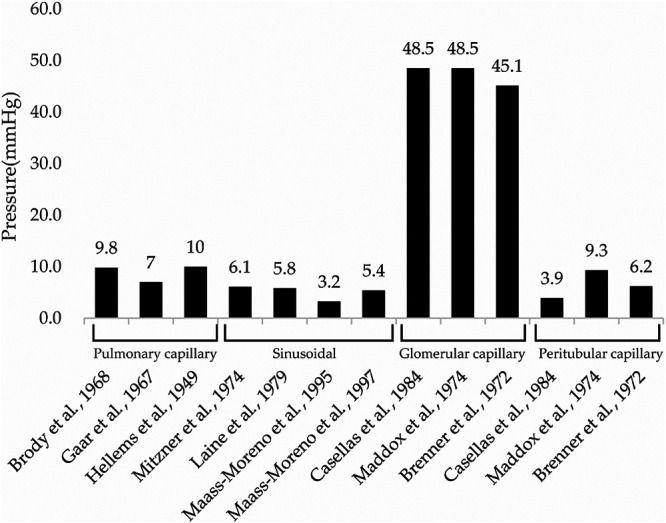
Pulmonary capillary pressure in the lung, sinusoidal pressure in the liver, glomerular capillary pressure in the renal cortex, and peritubular capillary pressure in the renal medulla. Data in this figure were obtained from ten studies.^31–40^ Pulmonary capillary pressure was measured by the following methods: 1) Lung lobes removed from mongrel dogs were perfused with blood from donor dogs and measured by a transducer,^31^ 2) the lower left lobe of lungs removed from mongrel dogs were measured by an isogravimetric method,^32^ and 3) pulmonary capillary wedge pressure was measured from a resting human.^33^ Sinusoidal pressure was measured by the following methods: 1) estimated by occluding the portal vein in anesthetized dogs,^34^ 2) interstitial pressure in anesthetized mongrel dogs was measured from polyethylene capsules implanted in the liver and described as an approximation of sinusoidal pressure,^35^ and 3) using a micropipette pressure-measuring system in anesthetized New Zealand White rabbits.^36,37^ Glomerular capillary pressure and peritubular capillary pressure were measured by the following methods: 1) kidneys removed from Sprague-Dawley rats were perfused at 100 mmHg and measured with micropressure measuring apparatus,^38^ 2) direct measurement by penetration from anesthetized squirrel monkeys,^39^ and 3) direct measurement by penetration of anesthetized Munich-Wistar rats.^40^ All pressures are displayed as mean values from each study and labeled above each bar. Figure 5 was created using Excel version 2202 and Adobe illustrator 2022.

Organs that are in contact with the external environment take up essential substances and excrete unwanted substances; however, bacteria and toxins can inadvertently be taken up at the same time. To protect organisms from external bacteria and toxins, their organs have well-developed immune functions.^29,30^ For instance, the lungs take up oxygen while discharging carbon dioxide, and contain both innate and adaptive immune cells.^29^. Similarly, the liver both absorbs and excretes substances from/into the intestinal tract via the enterohepatic circulatory system, and is therefore continuously exposed to antigenic stimuli including exogenous pathogens from the intestinal tract, dietary components, and foreign biological substances such as drugs and toxins.^30^ Although the lungs and liver have well-developed immune functions and rich blood flow, they are both low-pressure organs (Figure 6);^31–37^ this characteristic is hypothesized to facilitate interactions with foreign substances to allow leukocyte function. This hypothesis is consistent with the findings of our previous study, in which we demonstrated that NETs form in the lungs and liver of mice with endotoxin shock.^19^ Although pulmonary, sinusoidal, and peritubular capillaries have low perfusion pressure, glomerular capillaries have high perfusion pressure (Figure 6),^31–40^ suggesting that the kidney is a high-pressure organ. However, at the sub-organ level, the renal cortex is associated with high pressure and the renal medulla is associated with low pressure. Indeed, approximately 1 L of blood flows into the kidneys every minute, with 90% of blood flowing through the renal cortex,^41^ which filters out unwanted toxins as primary urine; in contrast, the medulla reabsorbs essential substances. In this study, no significant changes in citH3 quantity were observed in the renal cortex of any group (Figure 3, 5a), likely because the renal cortex is a high-pressure organ specialized for filtering urine.

Unlike the renal cortex, the renal medulla absorbs essential substances that constitute around 99% of the primary urine (100 L) produced by the glomerulus per day. Glomerular capillaries have pores with an approximately 60-nm diameter that rapidly filter out molecules <20 kDa, while partially or completely filtering out larger molecules. Previous studies reported that LPS accumulates in the proximal tubules,^42^ whereas damage-associated molecular patterns (DAMPs), such as histones, are filtered out by the glomerulus. The receptors of these molecules, Toll-like receptors 2 and 4, are located in the tubular epithelium and can cause inflammation upon binding.^43,44^ Peritubular capillaries wrap around the tubules of the renal medulla and reabsorb substances from the primary urine. The renal medulla has a highly developed immunity, largely due to the presence of many antigen-presenting dendritic cells in the peritubular interstitium.^23^ Accordingly, the renal medulla can induce high chemokine levels^24^ to prevent DAMPs and LPS from re-entering the peritubular capillaries during advanced sepsis. Here, we found that LPS increased citH3 in the renal medulla (Figures 4a, b; 5b), as hypothesized. Thus, we believe that the strong immune function of the renal medulla, as well as its low pressure, may facilitate the accumulation of NETs. Furthermore, we found that rTM suppressed citH3 (Figures 4b, c; 5b), suggesting that rTM can effectively inhibit NETs in the renal medulla. One limitation of this study is that the effect of rTM was explored only with regard to neutrophils, although it can bind to other immune cells. Although an endotoxin shock model was used, this study focused on renal injury, and only the effect on renal NETs on rTM was evaluated. Therefore, it is possible that other immune effectors not targeted by rTM may contribute to kidney injury after LPS administration. Indeed, this study did not reveal whether NETs formed in the kidney, but instead focused on whether rTM could suppress NETs in the kidney following induction by LPS.
